# Giant Gastric Antral Ulcer Developing After Short-Term NSAID Administration

**DOI:** 10.4021/gr382w

**Published:** 2011-11-20

**Authors:** Tateki Yamane, Takayuki Ishii, Akira Umeda, Akihiko Takeda, Hitoshi Shimao, Toshio Morisane

**Affiliations:** a Division of Gastroenterology, Department of Internal Medicine, The International University of Health and Welfare, Shioya Hospital, Japan; b Sanikukai Family Clinic, Japan; c Division of pulmonology, Department of Internal Medicine, The International University of Health and Welfare, Shioya Hospital, Japan; d Department of Surgery, The International University of Health and Welfare, Shioya Hospital, Japan

**Keywords:** Giant gastric ulcer, NSAID-induced gastric ulcer, Acute gastric ulcer

## Abstract

A 61-year-old man took loxoprofen sodium hydrate, a prodrug NSAID, for acute upper respiratory infection for 5 days, developed melena 2 days later, and was admitted to our hospital. Upper gastrointestinal endoscopy revealed a giant and deeply undermined ulcer mainly in the greater curvature of the antrum, which occupied halfway around the lumen. His medical history was unremarkable. He was negative for *Helicobacter pylori* infection, and was diagnosed with NSAID-induced acute gastric ulcer in the absence of other causes of gastric ulcer. Giant gastric ulcers, as in this patient, are rare. Moreover, deeply undermined or huge gastric ulcers sometimes develop during the long-term administration of NSAIDs, but very rarely after their short-term administration, which prompted us to report this case.

## Introduction

The two main causes of peptic ulcers are *Helicobacter pylori* (Hp) infection and NSAID use. Unlike Hp-induced gastric ulcers, NSAID-induced gastric ulcers occur mainly in the gastric antrum, and are characterized by being multiple in numbers, irregular in shape, deeply undermined or huge. However, deeply undermined or huge gastric ulcers usually develop during the long-term administration of NSAIDs. A 61-year-old man with no medical history took loxoprofen sodium hydrate (loxoprofen), a prodrug NSAID, for acute upper respiratory infection for 5 days, developed melena 2 days later, and was admitted to our hospital. Upper gastrointestinal endoscopy revealed a giant and deeply undermined ulcer mainly in the greater curvature of the antrum, which occupied halfway around the lumen. He was negative for Hp infection, and was diagnosed with NSAID-induced acute gastric ulcer in the absence of other causes of gastric ulcer. We report this case because giant gastric ulcers, as in this patient, are rare, and such gastric ulcers develop very rarely after short-term NSAID administration.

## Case Report

**Figure 1:**
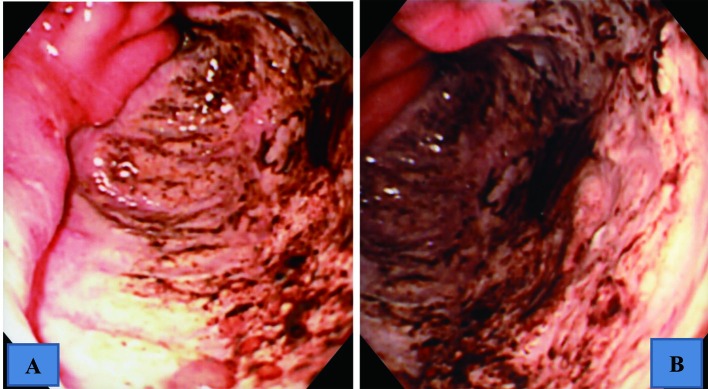
Upper gastrointestinal endoscopic findings showing a giant and deeply undermined ulcer mainly in the greater curvature of the antrum, which occupied halfway around the lumen. A: intermediate view. B: near view.

**Figure 2:**
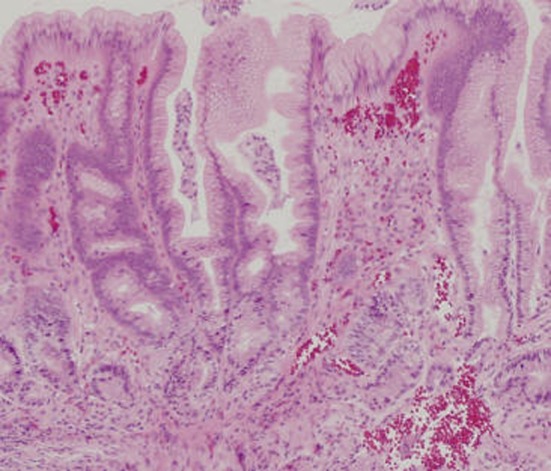
Histopathological findings in a biopsy specimen (HE staining) showing eroded pyloric gland tissue with no specific features.

**Figure 3:**
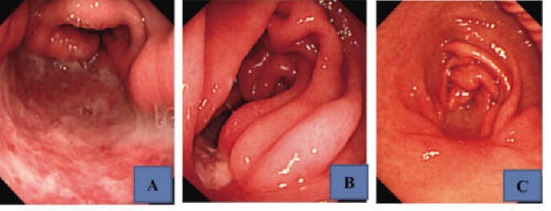
Upper gastrointestinal endoscopic findings after the start of administration of a PPI. A: At 1 week, the ulcer had somewhat reduced in size. B: At 4 months, the ulcer remained open, causing a marked deformity of the antrum. C: At 6 months, the ulcer had scarred and the antral deformity had improved.

A 61-year-old man took an antitussive drug (benproperine hydrochloride, 60 mg/day) and loxoprofen (60 mg/day), prescribed by a local physician, for 5 days because of acute upper respiratory infection. He developed epigastric pain the next day, and melena one day later. On the same day, he visited and was admitted to our hospital. He had no previous history of illness or smoking, and was a social drinker. He was not taking regular medication. Physical examination revealed anemia and epigastric tenderness. Laboratory tests showed a slight inflammatory reaction, with a CRP of 1.24 mg/dl, and a reduction of hemoglobin to 7.7 g/dl. Added examinations performed afterwards showed that he was negative for anti-Hp antibody, and his serum gastrin and pepsinogen I levels were within normal limits, at 87.2 ng/ml and 97.6 pg/ml, respectively. Upper gastrointestinal endoscopy revealed no abnormalities in the esophagus, duodenal bulb, or descending part of the duodenum, but demonstrated a giant and deeply undermined ulcer with blood clots in its base mainly in the greater curvature of the antrum, which occupied halfway around the lumen (Fig. 1 A, B). The rapid urease test was negative, and Hp was not found histopathologically. Histopathological examination of biopsies from the ulcer margin showed eroded pyloric gland tissue with no specific features (Fig. 2). Abdominal CT revealed no evidence of gastric perforation, and he was followed-up with a proton pump inhibitor (PPI) administration. Subsequently, his symptoms improved, and, on re-endoscopy 1 week later, the ulcer had somewhat reduced in size, and its base was almost clear (Fig. 3 A). He was discharged, continued on the PPI, and endoscopically followed-up every 2 months. The ulcer was reduced in size, but exhibited delayed healing, and remained open at 4 months, causing a marked deformity of the antrum (Fig. 3 B). At 6 months, scarring of the ulcer was observed, and the antral deformity had improved (Fig. 3 C). Although the PPI administration was discontinued, no ulcer recurred.

## Discussion

This patient developed a giant gastric antral ulcer after taking an NSAID for a short period of time. He was negative for Hp infection, and was diagnosed with NSAID-induced acute gastric ulcer in the absence of other causes of gastric ulcer. Two mechanisms are reportedly involved in acidic type NSAID-induced gastric mucosal injury in a synergistic manner: in one mechanism, NSAIDs acquire the ability to permeate cell membranes in an acidic environment and accumulate in epithelial cells, resulting in metabolic disturbances, leading to cellular disruption [1]. In the other mechanism, NSAIDs inhibit cyclooxygenases, resulting in a decrease in the production of endogenous prostaglandin (PG), as the major protective factor in the gastric mucosa, leading to mucosal injury [2]. Loxoprofen, an NSAID widely used in Japan, is a prodrug which exerts its action after absorption into the body; therefore, it should rarely cause direct damage to the gastric mucosa. However, its common use in clinical practice has caused many patients to develop NSAID-induced gastric ulcers [3]. It has been noted that a prodrug NSAID also reduces PG production, and the reflux of its active component (excreted from the liver into the bile) from the duodenum into the stomach is involved in the mechanism of gastric mucosal injury [4].

NSAID-induced gastric mucosal injury may occur at any point after the start of administration [5]. Studies in Japan have reported that the morphology of NSAID-induced gastric mucosal lesions may vary according to the elapse of time since their development, and they are classified into acute type and chronic type, depending on whether they develop within 2 weeks or at 4 weeks later after NSAID ingestion [6]. Lesions, whether acute type or chronic type, commonly occur in the antrum. Acute type lesions tend to be multiple irregular or geographic, shallow ulcers or erosions, whereas chronic type lesions are characterized by being multiple small ulcers or deeply undermined or irregular, large ulcers [6, 7]. The present case is rare in that the ulcer was acute type on clinical course, but so huge as not to have been seen in chronic type ulcers. Although the patient did not complain of epigastric pain during loxoprofen administration, the lesion had developed during that time, and, apparently, the pain had been controlled by loxoprofen. We speculate that multiple, geographic acute type ulcers, as characterized above, worsened during short-term ingestion of loxoprofen and rapidly coalesced to form a very huge ulcer.

Factors other than NSAID use may have been involved in the development of the gastric ulcer in this patient. However, they could not be elucidated, as the measured serum levels of gastrin and pepsinogen I excluded a hyperacidic state, no concomitant drugs other than an antitussive agent were administered, and no recurrence was observed despite PPI discontinuation after ulcer healing.

In conclusion, we reported a case of giant gastro-antral ulcer developing after short-term prodrug NSAID ingestion. Although this case is rare, side effects, such as gastrointestinal injury, should always be borne in mind even when administering prodrug NSAIDs for a short period of time.
